# Integration of precision medicine into the dental care setting

**DOI:** 10.3389/fdmed.2024.1398897

**Published:** 2024-08-21

**Authors:** Larissa Steigmann, Željka Perić Kačarević, Jessica Khoury, Katalin Nagy, Magda Feres

**Affiliations:** ^1^ Department of Oral Medicine, Infection, and Immunity, Division of Periodontology, Harvard School of Dental Medicine, Boston, MA, United States; ^2^ Department of Anatomy, Histology, Embryology, Pathology Anatomy and Pathology Histology, Faculty of Dental Medicine and Health Osijek, J.J. Strossmayer University of Osijek, Osijek, Croatia; ^3^ Department of Oral Biology, The Goldschleger School of Dental Medicine, Faculty of Medicine, Tel Aviv University, Tel Aviv, Israel; ^4^ Department of Oral Surgery, Faculty of Dentistry, University of Szeged, Szeged, Hungary

**Keywords:** precision medicine, salivary testing, biomarkers, primary care, point-of-care, periodontal diseases

## Abstract

This narrative review aims to discuss the incorporation of novel medical concepts and tools into dental practice, with the goal of improving early diagnosis and exploring new personalized treatment options for oral pathologies, such as caries and periodontitis. Preventative dental approaches concentrate on the timely detection of oral infections and the integration of biomarker analysis to recognize pathogenic changes at early stage of disease. Likewise, periodic monitoring after the treatment is relevant to ensure the balance in the oral biofilms and prevent relapse. Additionally, more attention has shifted towards the contributing factors to disease development, such as essential nutrients. Sufficient levels of vitamin C, vitamin D and zinc pre- and post-operatively are employed to boost immune function and reduce the risk of postoperative infections. Omega-3 fatty acids, melatonin, and antioxidants like vitamin E, which have anti-inflammatory properties, are utilized to help minimize excessive inflammation and promote faster recovery. The data presented in this manuscript emphasize the crucial integration of innovative healthcare concepts and tools into dental practices. By adopting a more holistic view of the patient, clinicians can tailor treatments to each individual's predispositions, lifestyle, and oral health conditions. This review also highlights the potential of salivary biomarkers and point-of-care technologies in enhancing early diagnostic accuracy and personalizing treatment. Bridging the gap between oral and systemic health is the most effective approach to improving patient quality of life. These findings underscore the importance of continued interdisciplinary collaboration in dentistry.

## Introduction

As the medical and dental fields evolve towards a new paradigm focused on prevention and minimally invasive procedures, both clinicians and patients increasingly advocate for care strategies that are both individualized and preventative. The adoption of telemedicine for continuous monitoring and non-invasive preventive interventions is already supporting this shift. Moreover, as the associations between system diseases and oral health gain more relevance, dental practice gains greater relevance. Furthermore, the recognition of the link between genetic predisposition, lifestyle choices and environmental or dietary factors has been a major aspect of scientific research, addressing the needs of patients based on their epigenetics, biomarkers and socio-economic characteristics with a potential to minimize diagnostic errors and improve treatment outcomes (1).

Oral health-related quality of life (OHRQoL) is an integral part of overall health and well-being. It is a multidimensional construct, including subjective evaluations of the individual's oral health, functional and emotional well-being, expectations and satisfaction with care, and self- perception. Recognized by the World Health Organization (WHO) as a crucial component of the Global Oral Health Program since 2003, the assessment of OHRQoL and extensive research in this area facilitate greater patient involvement in medical treatment, the integration of new approaches, and the monitoring of external factors influencing treatment outcomes (2).

**Figure 1:**
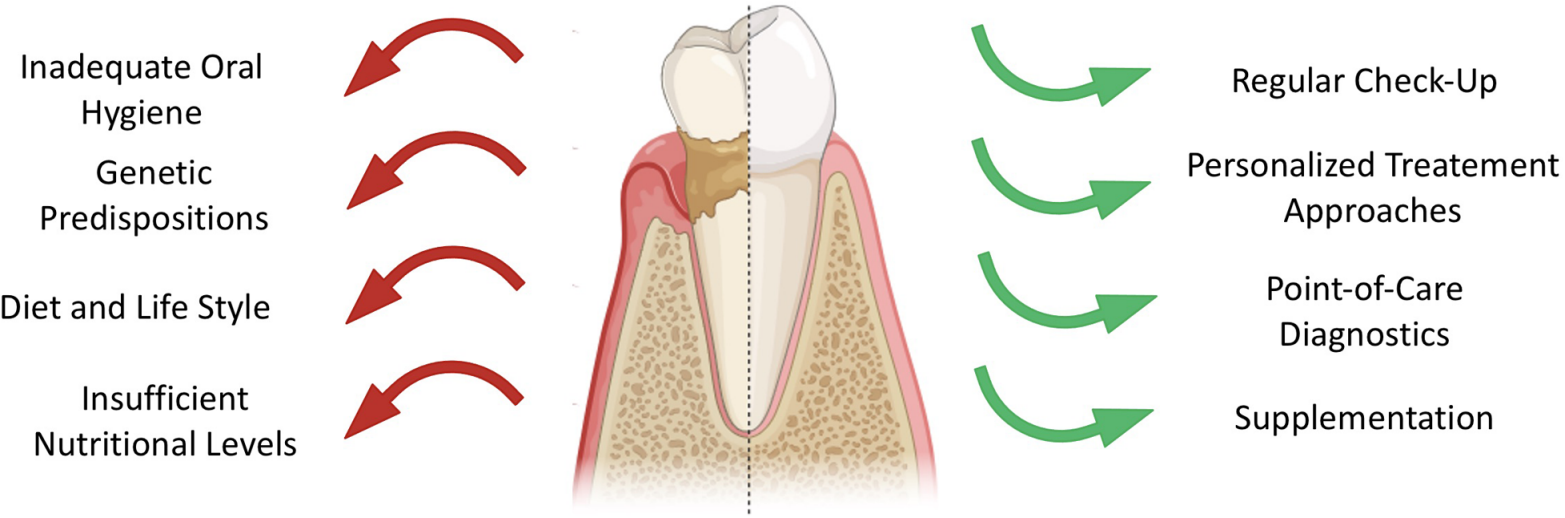
Overview of factors contributing to decreased oral health (left, red arrows) and factors enhancing dental health (right, green arrows).

Over the years, the dental approaches have shifted towards preventive measures and timely detection of oral infections. Yet, caries and periodontitis are still the major opponents of the maintenance of oral health (3, 4). These conditions result from dysbiosis in microbial biofilms and host factors, with dietary habits and hygiene playing as main drivers in disease outbreak and progression, as illustrated in Figure 1. Differential analysis between oral pathologies aids in implementing preventative measures and improvement of dental treatment (3, 5).

This narrative review aims to discuss the incorporation of novel medical concepts and tools into dental practice, with the goal of improving early diagnosis and exploring new personalized treatment options for oral pathologies, such as caries and periodontitis.

### Salivary screening for biomarkers

Understanding the field of dysbiosis and host- microbe interactions requires expanding our understanding beyond specific pathogens. The role of beneficial bacterial species and how they interact with each other is crucial (6). Host biomarkers also play a very important role on oral ecology, such as receptors for microbiome metabolites, lipid mediators of inflammation, chemokines, cytokines, enzymes, and other proteins. Exploring protein biomarkers not only provides insights into the pathogenic processes but also establishes the groundwork for precision medicine (6).

As previously mentioned, precision medicine demands a comprehensive system approach to integrate multi-level data in the search for accurate and reliable biomarkers of disease activity and novel therapeutic targets, with the aim to improve the management of chronic conditions across all medical domains (6). Biomarkers, measurable substances, serve as valuable data resources for indicating physiological health, screening pathological processes, and assessing responses to therapeutic intervention (6).

Numerous studies were carried out to analyze the relationships between saliva composition and oral diseases including periodontitis (7–10), carious lesions (11), and oral cancer (12, 13). These studies have shown that any shift in oral microbial biofilm and subsequent inflammatory response are reflected in saliva. As a result, saliva has been proposed as a potential medium for disease detection (7), even during early pathogenic changes prior irreversible tissue damage (14). Furthermore, saliva analysis has shown promises in monitoring disease progression (15) and evaluating responses to treatment strategies (15). Utilizing saliva as a diagnostic medium exerts several advantages; its collection is simple and painless, enabling for large quantities to be obtained without posing complications for the patients. Additionally, saliva demonstrates high durability (16).

Within the context of periodontitis, a nuanced understanding reveals three distinct phases: inflammation, connective tissue degradation, and bone resorption. Throughout each phase, the identification of specific biomarkers serves as a diagnostic compass, providing a comprehensive understanding of the patient's current stage of pathologic breakdown (17). The traditional diagnostic methods solely often fail to accurately reflect current disease activity and to identify high risk individuals (18). Hence, the meticulous monitoring of biomarkers stands as a pivotal element, emphasizing the significance of preliminary testing to inform the selection of supplementation dosage and duration. A recent systematic review highlights several biomarkers, including macrophage inflammatory protein-1 alpha (MIP-1α), IL-1β, IL-6, and MMP-8 (matrix metalloproteainase-8), as promising indicators for diagnosing periodontitis (19). Integrating clinical measures with assessments of pathogen and cytokines levels can yield a diagnostic sensitivity of up to 74% for predicting periodontitis progression (20). Future studies should focus on determining the optimal combination of biomarkers that would allow for the early diagnosis of periodontitis, as well as those that could be used to monitor treatment or serve as endpoints for treatments.

Salivary biomarkers can be an effective tool also in detecting caries in earlier stages and assessing a patient's caries risk. These biomarkers include nitrite, nitrate, immunoglobulin A, mucin, histatin and proline- rich proteins. A recent systematic review has demonstrated that nitrite levels in saliva serve as a reliable biomarker for dental caries in children (21). However, further research in this area is necessary. Additionally, the application of Artificial Intelligence (AI) could enhance the capability of salivary biomarkers to accurately diagnose and manage several oral diseases, including caries and periodontitis (22).

### Oral-systemic axis

Over the past decades, there has been a growing recognition of the bidirectional connection between oral health, particularly periodontal health, and overall systemic health. This has led to a heightened awareness among physicians regarding the significance of prioritizing oral health alongside systemic health, given that oral cavity serves as a significant reservoir of microorganisms (23). Special high-risk groups emphasize the interplay between oral and systemic health. The suggested mechanisms that facilitate the connection between oral and systemic health involve predisposing and precipitating factors. These encompass genetic elements like gene polymorphisms, environmental influences such as stress and habits like smoking, and dietary choices featuring high-fat or heavily processed foods. Furthermore, medications, microbial dysbiosis, bacteremia, and changes in the host immune response contribute to mediating this relationship (24).

Specifically, periodontitis and periodontal pathogens have been associated with diabetes mellitus, metabolic syndrome, obesity, eating disorders, liver disease, cardiovascular disease, Alzheimer disease, rheumatoid arthritis, adverse pregnancy outcomes, and cancer (24–26).

#### Diabetes mellitus

Diabetes mellitus is a well- established risk factor for periodontitis (8), with some experts even considering it as “the sixth complication of diabetes mellitus” (27). Additionally, active periodontitis has been shown to adversely impair glycemic control, resulting in hyperglycemia that produces advanced glycation end products (AGE). In turn, AGEs stimulate the overproduction of pro- inflammatory cytokines such as IL-6, IL-1 and Tumor Necrosis Factor alpha (TNF-α). As a consequence, the presence of AGEs in tissues impairs wound healing by disrupting collagen turnover and promoting an excessive neutrophil response to periodontal bacteria. Maintaining glycemic levels within the normal ranges is crucial for preventing periodontal complications (28). For example, studies conducted by Preshaw et al. and Steigmann et al. revealed a marked rise in the risk of periodontal-related issues with higher levels of capillary blood glucose (29, 30). Additionally, periodontal therapy has been shown to have a positive effect in reducing insulin resistance leading to improvement in glycemic control (28).

#### Coronary heart disease

*Porphyromonas gingivalis* induces vascular permeability by degrading endothelial adhesion molecules via gingipain-dependent mechanisms. This activity can elevate platelet aggregation, enhance leukocyte adhesion, and increase secretion of pro-inflammatory cytokines, all contributing to the development and progression of atherosclerosis, the primary cause of cardiovascular disease. Immunolocalization studies have identified *P. gingivalis* in 42% of infectious agents present in atherosclerotic plaques. These microorganisms, located within unstable plaque regions, pose risks such as plaque ulceration, thrombosis, and vascular cell apoptosis (31). Periodontitis results in elevated systemic levels of C- reactive protein, IL-1*β*, IL-6, IL-8, and TNF-*α* (32)*.* These systemic markers of inflammation are also considered as predictors of current and future cardiovascular events and disease (30). Untreated periodontitis is linked to elevated plasma levels of LDL, cholesterol and triglycerides, accompanied by a decrease in HDL concentrations (28).

#### Obesity

Cardiovascular disease symptoms and diabetes mellitus are part of the metabolic syndrome cluster, alongside obesity. Obesity results in an imbalance between increased inflammatory stimuli, reduced anti-inflammatory mechanisms, and persistent low-grade inflammation, triggering periodontitis and further obesity status. Obese patients are 50%–80% more likely to develop of periodontitis compared to non-obese individuals (33, 34).

#### Alzheimer disease

*P. gingivalis* has also gained attention for its implication in neurodegenerative disorders, as it can migrate from the oral cavity into the bloodstream into and then reach distal sites, such as the brain. This microbial translocation can lead to local inflammation and buildup of the hallmark signs of Alzheimer disease, including beta-amyloid deposits, tau fragmentation and tangles, and the breakdown of host protective molecules, like apolipoproteins, leading to neuron toxicity (24). Consistent with this, *P.gingivalis’* DNA and gingipains have been detected in brains of individuals with Alzheimer's disease, as well as in cerebrospinal fluid (35).

#### IL-1 gene polymorphism

Individuals with severe periodontal genotypes may exhibit susceptibility alleles more frequently. IL-1, a potent pro-inflammatory cytokine, plays a crucial role in the pathogenesis of numerous chronic human conditions, including cardiovascular, metabolic, and autoimmune diseases (36). In the context of periodontitis, the IL-1 genotype has been shown to regulate local inflammatory processes (9, 37) and conversely, individuals with stage III and IV periodontitis were found to have a higher likelihood of cardiovascular disease compared to those with stage I (adjusted odds ratio 3.59) (38).

#### Rheumatoid arthritis

Individuals with moderate to severe periodontitis are at a heightened risk of developing rheumatoid arthritis. There's a suggestion that periodontal disease could be a contributing factor in both initiating and sustaining the autoimmune inflammatory response characteristic of rheumatoid arthritis (39). Reviews of studies have shown that erythrocyte sedimentation rate and C- reactive protein levels may be raised in rheumatoid arthritis patients with periodontitis. Furthermore, serum antibodies targeting periodontal pathogenic bacteria, like *Porphyromonas gingivalis* have been found in the synovium. *P. gingivalis*’ presence may also worsen rheumatoid arthritis by triggering the production of anticitrullinated protein antibodies (40).

### Nutritional support

The importance of nutrition and its role in contributing to periodontal disease has been already described (41–43). While a balanced diet is crucial for maintaining periodontal health particularly, it is also essential for optimal tissue repair and immune function before and after periodontal procedures. Supplementation of specific nutrients that could be lacking may be necessary. Therefore, emphasis is placed on nutritional supplements for ensuring optimal regenerative treatment (44, 45). The supplemented nutrients and their role in oral health are summarized in Table 1.

**Table 1:** Summary of the supplement nutrients impacting periodontal health.

Nutrient supplement	Importance in Dental Health
Vitamin A	Preservation of mucosal membranes, salivary glands and teeth (46)
Vitamin B12	Contributing to mucosal wound healing (47)
Vitamin C	Pivotal in collagen synthesis; Antioxidative property (47)
Vitamin D	Promoting bone mineralization through enhancement of minerals absorption in intestines (48–50); Anti-inflammatory property via inhibition of pro-inflammatory cytokines secretion and promoting expression of anti-inflammatory cytokines (48)
Vitamin E	Antioxidative property; Promoting wound healing by preventing prostaglandins’ synthesis (47)
Iron	Linked to antioxidant enzymes (51)
Zinc	Antioxidative property; Reduction of gingival inflammation by decreasing sulcular epithelium permeability (52)
Calcium	Maintenance and promotion of calcified tissues development; Proper function of blood cells (47)
Omega- 3 fatty acids	Anti- inflammatory property; Bone repairing property (53)
Melatonin	Supporting new bone formation by promoting mesenchymal stem cells differentiation into osteoblasts and controlling phosphorous and calcium metabolism; Antioxidative property (54)

#### Vitamins

Vitamin A, a fat-soluble vitamin, is crucial for the preservation of the mucosal membranes, salivary glands, and teeth (46). The vitamin B complex family has an importance in cell metabolism, repair, and proliferation. Deficiency in vitamin B can lead to various diseases and symptoms; oral manifestations can be seen as angular cheilitis, glossitis and gingival bleeding. Zong et al.'s recent study revealed a reverse correlation between the severity of periodontitis and serum vitamin B12 levels. Increased levels of vitamin B12 were associated with a decrease in the clinical parameters of periodontitis since vitamin B12 is known to contribute towards mucosal wound healing and facilitating bone health, both of which are necessary for recovery in cases of periodontitis (47).

Vitamin C, also known as ascorbic acid, is a pivotal element for collagen synthesis and preventing oxidative stress. Its deficiency results in Scurvy disease, which has periodontal hallmarks as well, notably bleeding, inflamed and painful gums. Given its beneficial impact on periodontal health, several *in vitro* studies suggest applying vitamin C in coatings or gels to promote osseointegration of dental implants and improve post- surgical healing. Additionally, for smokers, the application of ascorbic acid can be utilized to reduce the typical periodontal tissue breakdown in these patients (47).

Vitamin D also contributes to oral health preservation by promoting bone mineralization through enhanced absorption of calcium, magnesium, zinc and phosphate in the intestine. Deficiencies in vitamin D have been associated with chronic diseases such as periodontitis and diabetes and with tooth decay (48–50), Extensive studies have explored the anti- inflammatory properties of vitamin D inflammation; vitamin D inhibits the production of pro-inflammatory cytokines like IL-6 and IL-8, which are involved in acute inflammation, while promoting the expression of the anti-inflammatory cytokine IL-10. Furthermore, studies have revealed lower levels of vitamin D levels in patients with periodontitis compared to healthy patients, and its supplementation has been shown to improve periodontal parameters (48). These findings highlight the regulatory role of vitamin D in both the innate and adaptive immune system and its crucial impact on inflammation in the body, particularly in dental aspects (55–57).

Vitamin E plays a crucial role in oral health maintenance by inhibiting the reaction of free radicals, thereby aiding in membrane stabilization. Beyond its antioxidant property, vitamin E has demonstrated positive effects on periodontal health by intruding on the prostaglandins’ synthesis, which is helpful in reducing inflammation level and improving wound healing (47).

#### Minerals

In a recent study conducted by Chakraborty et al., it was revealed that iron-deficiency anemia is linked to a decline in antioxidant enzymes, leading to increased oxidative stress and a worsening of periodontal diseases (51).

Zinc is reported to decrease the sulcular epithelial permeability by inhibiting the leukocyte activity, thus decreasing gingival inflammation via decreasing gingival fluids. Furthermore, it was noticed that zinc acts to counteract reactive oxygen species and bacterial toxins, promoting healthy periodontium (52).

Calcium is indispensable for the maintenance and development of calcified tissues like bones and teeth. Additionally, it plays a crucial role in the proper functioning of blood cells. A deficiency in dietary calcium can also affect periodontal health. Co-supplementation of calcium and vitamin D is frequently employed and has been shown to have a beneficial impact on the outcomes of periodontal therapy. Research has demonstrated that the local administration of calcium, in the form of hydroxyapatite, improves the osseointegration of dental implants (47).

The protective effects of fluoride against tooth decay have long been recognized. Fluoride works by strengthening enamel and cementum through the formation of fluoroapatite, while also inhibiting bacterial growth and adhesion, effectively preventing caries. Consequently, various forms of topical fluoride, such as mouth washes, toothpaste, gels, foams, and varnishes, are commonly used as preventive measures against dental caries. Furthermore, recognizing its benefits, fluoride has been integrated into different restorative materials like glass ionomers. Systemic fluoride can be administered through water, milk, or capsules, with supplementation ranging from 0.25 to 1 mg per day depending on the existing fluoride concentration in drinking water (47).

#### Omega-3-fatty acids and melatonin

Omega-3 fatty acids and melatonin are recognized for their anti-inflammatory and bone repairing properties. By inflammation control, omega-3 fatty acids indirectly reduce the excessive breakdown of connective tissue and bone, while also enhancing the blood flow to affected areas. Also, omega-3 fatty acids have an impact on prostaglandin production, thereby altering bone metabolisms (53). On the other hand, melatonin is a hormone which regulates various factors such as calcitonin, growth factors and corticosterone, all of which are involved in bone metabolisms. Moreover, melatonin promotes mesenchymal stem cells differentiation into osteoblasts and can regulate phosphorus and calcium metabolism, thus supporting new bone formation and repair. Melatonin's antioxidant properties enable it to neutralize oxidative stress in the oral cavity, which has been linked to periodontal disease (54). Therefore, supplementation with omega-3 fatty acids and melatonin has been reported to enhance outcomes in regenerative dentistry, plaque score improvements, reduction of gingival inflammation and enhancement of periodontal healing (53, 54).

## Point-of-care technologies

### Patient involvement

Generally, personalized medicine not only concentrates on the recognition of associations between systemic diseases and contributing factors but also aims to tailor treatments based on an individual's unique characteristics and preferences. Therefore, by involving patients in decision- making discussions regarding medical approaches, clinicians acknowledge the importance of autonomy to make decisions about their healthcare and highlight the effects on their well- being. Furthermore, patients often rely on information from different sources when being confronted with unfamiliar problems. By providing support during the decision- making processes, clinical staff have the opportunity to be transparent about the complexity of the medical situations and inform the patients about genetic testing, biomarkers, treatment options, and potential risks or benefits. This allows the engagement in discussions resulting in a clear understanding of potential treatment options and the implementation of lifestyle modifications and required habit changes. Throughout this process, clinicians are able to address associated uncertainties and promote trust in patient-centered care, which is more likely to result in the adhesion to the treatment plan and, thus, improved patient satisfaction (58, 59).

### Periodical monitoring

Regular testing of biomarkers may be beneficial for patients who are at risk for deficiencies or pathogenicity. Constant monitoring allows risk stratification of patients into low to high categories, considering both modifiable and nonmodifiable risk factors, as well as immune-metabolic indicated by biomarkers. The oral phenotype, determined by genetic, environmental, and microbial factors, is reflected by tissue integrity and immune functions that control the pathogenicity of the oral biofilms. Integrating gene biomarkers with conventional risk factors in a personalized medicine approach enables population stratification, aiding in resource allocation for preventive dentistry (60).

**Figure 2:**
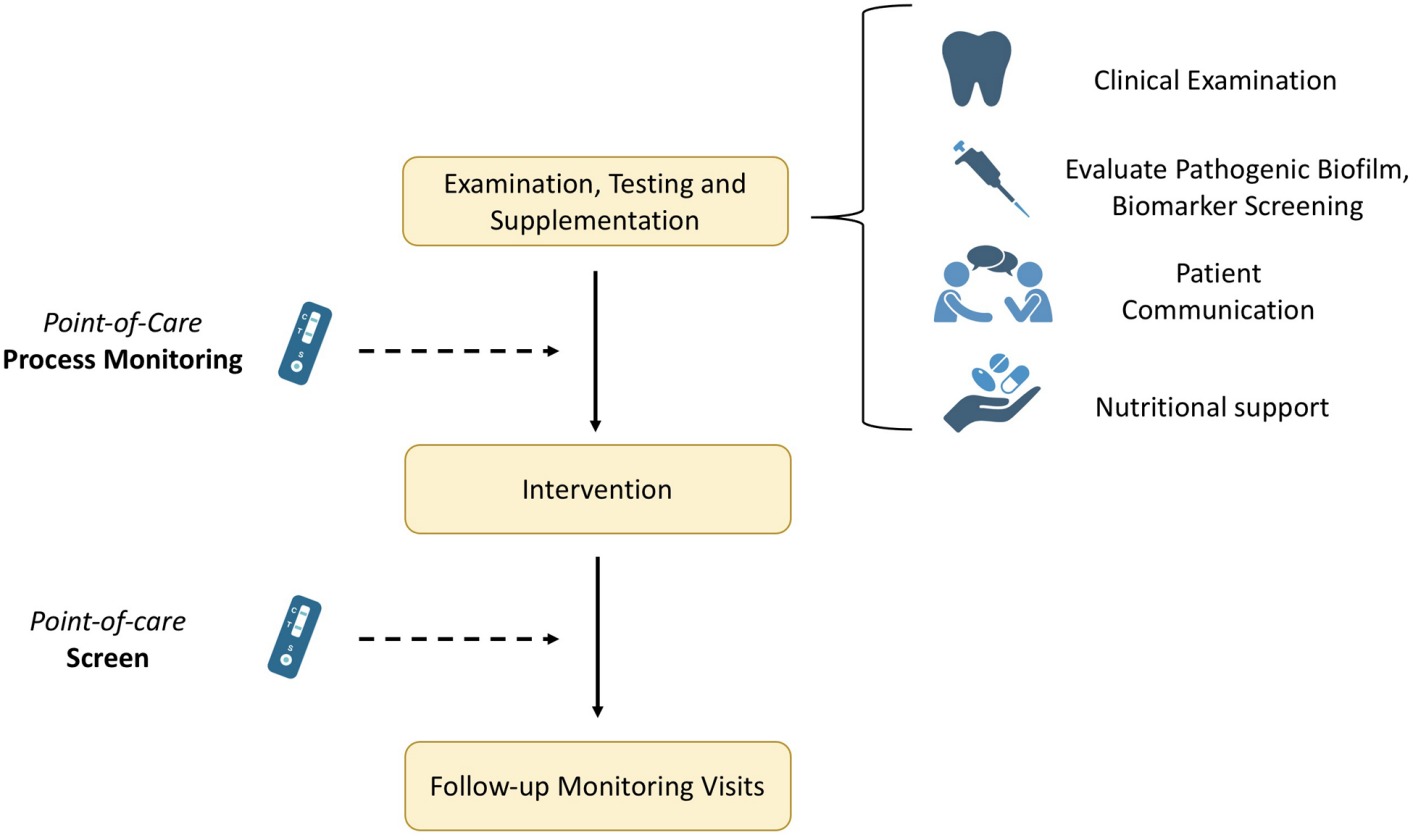
Exemplar workflow of a dental treatment plan with integrated personalized approach.

Apart from the rapid diagnosis, novel approaches allow for the implementation of various tests over an extended period to monitor progress or assess healing parameters, thereby preventing complications or relapse (Figure 2). When supplementation is needed, the periodic monitoring of biomarker levels is crucial to follow any fluctuation in serum concentrations. Thus, these novel testing methodologies offer a more advanced approach via the point-of-care testing and the optimization of the whole process. Therefore, mapping the levels before and after e.g., a surgical intervention is easier and with minimized outlay. Likewise, the importance of regular preventative visits is highlighted in literature. Giannobile et al. investigated insurance claims for 16 years for 5,117 adults retrospectively for tooth extraction events and reported significantly reduced tooth loss rates associated with increased preventive visits (60). Hence, emphasizing the importance of patient awareness, enhanced regular check-ups, as well as integration of marker screening and nutritional monitoring in patients’ treatment approaches to support improved clinical outcomes.

### Point-of-care testing and triaging

**Figure 3:**
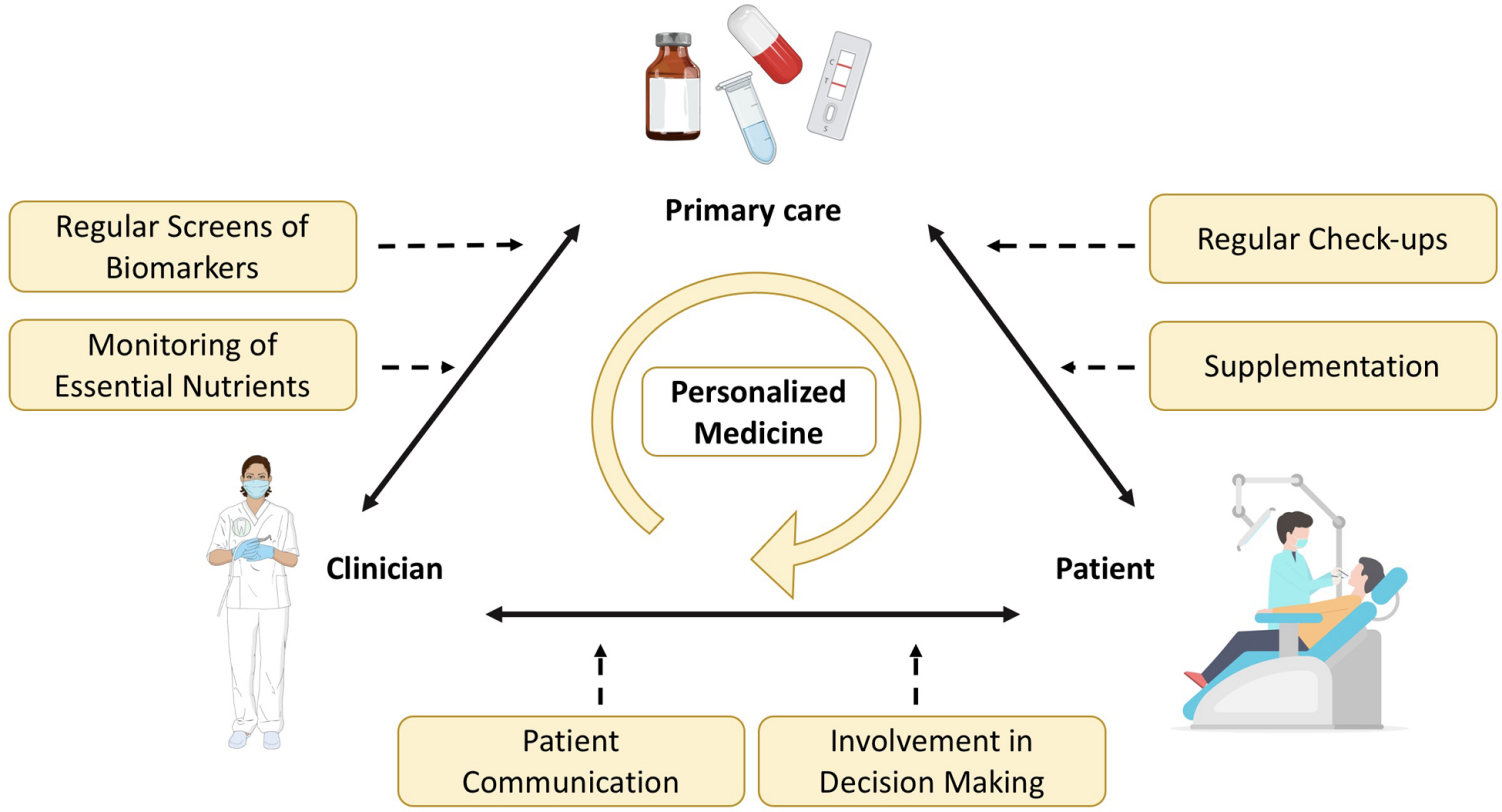
Overview of personalized medicine approaches in the dental health practice.

The development of new point-of-care technologies has revolutionized the way clinicians can test marker levels directly in their offices, eliminating the need for external laboratories involvement. This advancement not only allows for rapid diagnostics but also facilitates the formulation of personalized treatment plan (Figure 3). Furthermore, reducing chair time in dental offices improves efficiency and productivity for staff while potentially enhancing the overall patient's dental experience, as patients commonly associate dental procedures with discomfort. By minimizing chair time, patients are spared the inconvenience of scheduling additional appointments at external laboratories and then returning to the dental office for further consultations. Additionally, shorter chair time may contribute to more efficient scheduling and better outcomes. Instead, it can guide and triage the patient to any follow up or consultation in medical practice that are necessary post-screening. This interdisciplinary workflow, starting in the dental office, promotes pre-appointment preparation and excludes any additional testing reducing likelihood of missed cancer screening or skipped appointments for patients with limited time available. Ultimately, reducing chair time encourages patients to prioritize their oral health and ensures that they receive the necessary care promptly.

### Integrated primary care

Oral health is increasingly recognized as an integral part of general health, prompting the integration of dental care into primary healthcare services (Figure 3). This shift has put the spotlight on various risk factors such as lifestyle, systemic conditions, diet and smoking, which contribute to oral diseases. Integration strategies not only advocate to raise awareness about oral health but also seek to enhance individual's willingness to prioritize it. The integration of oral health into primary care has been implemented to reduce the burden of oral diseases within the communities and to promote health education and empowerment. For example, the Longwood Harvard Dental Center (HDC) has launched an innovative project known as the Nurse Practitioner-Dentist (NPD) model for Primary Care in a dental practice environment. Recognizing the pivotal role nurse practitioners play in providing comprehensive healthcare services to patients, such as physical examinations, annual wellness check- ups, and counseling on nutrition and tobacco cessation. The program's goal is to optimize the value of each patient encounter by improving outcomes and delivering more holistic care through the seamless integration of these services into the dental visit. Other strategies concentrate on building interdisciplinary networks, training non-dental care providers and implementing e-health technologies (61). The ultimate goal is to integrate oral health into overall health, acknowledging the relationship between oral and systemic well-being.

## Conclusion

This review emphasizes the crucial integration of innovative healthcare concepts and tools into dental practices. By adopting a more holistic view of the patient, clinicians can tailor treatments to each individual's predispositions, lifestyle, and oral health conditions. The data also highlights the potential of salivary biomarkers and point-of-care technologies in enhancing early diagnostic accuracy and personalizing treatment. Bridging the gap between oral and systemic health is the most effective approach to improving patient quality of life. These findings underscore the importance of continued interdisciplinary collaboration in dentistry.

## Data Availability

The original contributions presented in the study are included in the article/Supplementary Material, further inquiries can be directed to the corresponding author.
